# Decoction of Sage in Profuse Sweating

**Published:** 1867-10

**Authors:** 


### *Decoction of Sage in Profuse Sweating.*

M. Vignard, of Nantes, after the manner of Van Swieten, has successfully employed decoction of sage for the relief of profuse sweating. In the case of a man twenty-five years of age, who had suffered, from time to time, during many years from attacks of this kind, the remedy proved effectual. The sweating began suddenly between two and three o'clock in the morning all over the body, and was so profuse as to completely saturate the bed-clothes and to a considerable extent the mattress also. In consequence of the regularity of the attacks, sulphate of quinia was tried as an antiperiodic, but unavailingly; the perspiration regularly re-appeared, and without any apparent pathological cause. At length M. Vignard prescribed the following preparation: Take of chopped sage leaves, a large teaspoonful (*une forte princée*;) of water, six fluid ounces. Boil the sage for a minute or two in the water; let it stand to cool, then filter and sweeten to taste. From that time the perspiration ceased whenever the decoction was taken, re-appeared when it was omitted. M. Vignard suggested the use of this remedy in the colliquative sweating of phthisis.—*Journ. de Méd. de Nantes.*

